# Endothelial cell activation is attenuated by everolimus via transcriptional and post-transcriptional regulatory mechanisms after drug-eluting coronary stenting

**DOI:** 10.1371/journal.pone.0197890

**Published:** 2018-06-11

**Authors:** Zsolt Fejes, Zsolt Czimmerer, Tibor Szük, Szilárd Póliska, Attila Horváth, Enikő Balogh, Viktória Jeney, Judit Váradi, Ferenc Fenyvesi, György Balla, István Édes, József Balla, János Kappelmayer, Béla Nagy

**Affiliations:** 1 Department of Laboratory Medicine, Faculty of Medicine, University of Debrecen, Debrecen, Hungary; 2 Department of Biochemistry and Molecular Biology, Genomic Medicine and Bioinformatics Core Facility, Faculty of Medicine, University of Debrecen, Debrecen, Hungary; 3 Department of Cardiology, Faculty of Medicine, University of Debrecen, Debrecen, Hungary; 4 Department of Internal Medicine, Faculty of Medicine, University of Debrecen, Debrecen, Hungary; 5 Department of Pharmaceutical Technology, Faculty of Pharmacy, University of Debrecen, Debrecen, Hungary; 6 MTA-DE Vascular Biology, Thrombosis and Haemostasis Research Group, Hungarian Academy of Sciences, Debrecen, Hungary; Universita degli Studi di Napoli Federico II, ITALY

## Abstract

We previously found higher level of endothelial cell (EC) activation in patients who suffered from in-stent restenosis after bare-metal stenting compared to subjects who underwent drug-eluting stenting (DES) showing no complications. Here we investigated the potential transcriptional and post-transcriptional regulatory mechanisms by which everolimus attenuated EC activation after DES. We studied the effect of everolimus on E-selectin (SELE) and VCAM1 mRNA levels when human coronary artery (HCAECs) and human umbilical vein ECs were challenged with recombinant TNF-α (100 ng/mL) for 1–24 hours in the presence or absence of everolimus using 0.5 μM concentration locally maintained by DES. EC activation was evaluated via the levels of IL-1β and IL-6 mRNAs with miR-155 expression by RT-qPCR as well as the nuclear translocation of nuclear factor kappa beta (NF-κB) detected by fluorescence microscopy. To investigate the transcriptional regulation of E-selectin and VCAM-1, TNF-α-induced enhancer RNA (eRNA) expression at p65-bound enhancers in the neighboring genomic regions of SELE and VCAM1 genes, including SELE_-11Kb and VCAM1_-10Kb, were measured in HCAECs. Mature and precursor levels of E-selectin and VCAM-1 repressor miR-181b were quantified to analyze the post-transcriptional regulation of these genes in HCAECs. Circulating miR-181b was analyzed in plasma samples of stented subjects by stem-loop RT-qPCR. TNF-α highly elevated E-selectin and VCAM-1 expression at transcriptional level in ECs. Levels of mature, pre- and pri-miR-181b were repressed in ECs by TNF-α, while everolimus acted as a negative regulator of EC activation via inhibited translocation of NF-κB p65 subunit into cell nuclei, lowered eRNA expression at SELE and VCAM1 genes-associated enhancers and modulated expression of their post-transcriptional repressor miR-181b. Significant negative correlation was observed between plasma miR-181b and soluble E-selectin and VCAM-1 in patients. In conclusion, everolimus attenuates EC activation via reduced NF-κB p65 translocation causing decreased E-selectin and VCAM-1 expression at transcriptional and post-transcriptional level after DES.

## Introduction

The expanding application of drug-eluting stents (DES) has dramatically decreased the incidence of in-stent restenosis (ISR) compared to bare metal stents (BMS) [1]. These stents are covered with an anti-proliferative drug, such as everolimus that slows down the endothelialization in stented coronary arteries [1]. Early ISR is primarily caused by subsequent endothelium dysfunction and activation and uncontrolled neo-intimal proliferation [2, 3]. It has previously been reported that percutaneous coronary intervention (PCI) could cause endothelial cell (EC) activation accompanied with enhanced E-selectin [4] and vascular cell adhesion molecule-1 (VCAM-1) plasma concentrations [5, 6]. Both adhesion receptors are expressed on activated ECs stimulated by tumor necrosis factor α (TNF-α) or other inflammatory cytokines in large part via increased transcriptional regulation, and then involved in leukocyte migration to ECs [7]. Furthermore, BMS-induced restenosis was associated with higher soluble VCAM-1 and TNF-α levels in a clinical study [8]. Recently, our group has compared the effect of BMS and DES on the degree of endothelium and platelet activation in the light of incidence of ISR in stable angina patients [6]. We described that 20% of BMS subjects suffered from ISR developed after 1–3 months of intervention compared to DES individuals. Based on soluble E-selectin and VCAM-1 concentrations measured at 1 month follow-up samples, there was more EC activation in BMS patients with ISR compared to DES subjects without any complication [6].

Everolimus is an inhibitor of mammalian target of rapamycin (mTOR) and is currently used as an immunosuppressant to prevent rejection of organ transplants. However, this drug has an anti-proliferative effect via blocking the cell cycle in the G1 phase to inhibit proliferation, such as in vascular smooth muscle cells (VSMC) [9], and has recently showed a potent anti-inflammatory effect in neutrophils reducing the release of IL-8 and decreasing TNF-α-induced adhesion of neutrophils to ECs [10]. In parallel, rapamycin (sirolimus) antagonized VCAM-1 levels induced by TNF-α in human umbilical vein endothelial cells (HUVECs) via inhibiting mTORC2 activity and potentiated ERK1/2 [11]. However, no studies have investigated the potential transcriptional and post-transcriptional regulatory mechanisms by which everolimus can decrease EC activation.

MicroRNAs (miRNA) have recently been introduced as post-transcriptional fine regulators in various pathophysiological processes, such as in vascular disorders [12]. For instance, miR-133a, miR-155, and miR-126 have been connected with different cellular and inflammatory responses of the vessel wall acting as potential biomarkers in cardiovascular diseases [13, 14]. PCI-related ISR was also associated with altered miRNA expression, i.e. plasma miR-21 was overexpressed in subjects with ISR [15], while overexpression of endothelial miR-126 prevented vascular restenosis in a rat balloon injury model [16]. In addition, EC activation-dependent VCAM-1 and E-selectin were modulated by miR-181b [17].

To date, limited pieces of evidence are available about transcriptional and post-transcriptional regulatory mechanisms of E-selectin and VCAM-1 expression upon the development of enhanced EC activation as well as its inhibition by everolimus. Thus, we here analyzed E-selectin and VCAM-1 expression at transcriptional level in two types of EC cultures. In addition, we measured the mature and precursor forms of E-selectin/VCAM-1 post-transcriptional regulator miR-181b in EC cultures stimulated with TNF-α in the presence or absence of everolimus *in vitro*. Furthermore, the levels of miR-181b were also investigated and correlated with the concentrations of these related soluble adhesive receptors in plasma samples of patients who underwent BMS or DES implantation with or without ISR.

## Materials and methods

### Culturing endothelial cells with or without everolimus

Human coronary artery endothelial cells (HCAEC, Cell Applications Inc, San Diego, CA, USA) were cultured in ready-to-use MesoEndo Cell Growth Medium (Cell Applications) at 37°C, 5% CO_2_. In parallel, HUVECs were specifically isolated for this study and were removed from human umbilical veins by exposure to dispase and cultured in medium 199 (M199, Gibco, Grand Island, NY, USA) containing 15% fetal bovine serum (Gibco), antibiotic, antimycotic solution (1%, Sigma), heparin (5 U/mL, Merckle GmbH, Blaubeuren, Germany) and endothelial growth supplement (7.5 ug/mL, Sigma) as described in our previous study [18]. For subculturing, cell density was set to 5,000 cells per cm^2^ in both cell cultures.

HCAEC and HUVEC cells (3x10^5^/well) were treated in 6-well plates with recombinant TNF-α (100 ng/mL, Gibco) for 1–24 hours to generate cellular inflammatory conditions as an *in vitro* model of stent-induced EC inflammation. In parallel, the effect of everolimus on EC activation was studied using everolimus (0.5 μM, dissolved in DMSO, Sigma) in the presence of TNF-α for the same time period above. After treatment, cells were washed once with sterile Hanks’ Balanced Salt solution (Sigma), then lysed in 750 μL TRI reagent (Molecular Research Center INC, Cincinnati, OH, USA) and stored at -20°C before RNA isolation.

Total RNA was then extracted for the quantification of E-selectin and VCAM-1 mRNAs as well as miR-181b using RT-qPCR. The purity and the concentration of separated RNA samples were verified by a NanoDrop spectrophotometer (Thermo Scientific, Wilmington, DE, USA). Total RNA samples were stored at -70°C before quantitative analysis.

### RT-qPCR analysis of mRNAs, pre-miRNAs and pri-miRNAs

cDNA synthesis was performed with High-Capacity cDNA Reverse Transcription Kit (Applied Biosystems, ABI, Foster City, CA, USA) according to the manufacturer’s recommendation with minor modifications. Initial amount of RNA in case of ECs was 500 ng per reaction. Quantitative PCR was performed using LC-480 instrument (Roche Diagnostics GmbH, Mannheim, Germany) with LightCycler 480 SYBR Green I Master mix (Roche Diagnostics) and gene specific primers (10 μM, Integrated DNA Technologies Inc, IDT, Leuven, Belgium). The reactions were incubated at 95°C for 10 min, followed by 40 cycles of 95°C for 10 sec and 60°C for 1 min. For normalization, we used the reference gene RPLP0 (36B4). Sequences of these primers are listed in S1 Table.

### Measurement of soluble E-selectin and VCAM-1 levels in supernatants of EC cultures

The concentrations of E-selectin and VCAM-1 were determined in the supernatants of HCAEC cultures by using commercially available enzyme-linked immunoassays (ELISA) (R&D Systems, Minneapolis, MN, USA) according to the manufacturer’s instructions. Before performing ELISA, samples were centrifuged at 10,000 *g* for 1 min to obtain cell free supernatants.

### Investigation of the effect of everolimus on inflammatory response to TNF-α via measuring IL-1β and IL-6 expression in ECs

To determine if TNF-α-induced EC activation was modulated at transcriptional level by everolimus, HCAECs were treated with recombinant TNF-α (100 ng/mL) with or without everolimus (0.5 μM) for 1 and 4 hours, then IL-1β and IL-6 mRNA levels as sensitive inflammation biomarkers were quantified by RT-qPCR as shown above. Sequences of primers for IL-1β and IL-6 mRNAs are listed in S1 Table.

### Detection of nuclear factor kappa B (NF-κB) activation in ECs

The p65 staining was performed based on our previous publication [19]. Briefly, HCAEC cells were seeded onto sterile uncoated microscope slides at a density of 5 x 10^4^ cells/slide and cultured for 2 days. HCAECs were stimulated with TNF-α (100 ng/mL) for 1 hour in the absence or presence of everolimus (0.5 μM) or DMSO, and were then fixed with ice-cold methanol-acetone (50 v/v %) for 10 min. Non-specific antibody binding sites were blocked with FBS for 15 min. For primary labelling of NF-κB p65 subunit, rabbit anti-human p65 (100 μg/ml, Santa Cruz Biotechnology, AB_632037) was used followed by secondary staining with Alexa Fluor 488-conjugated goat-anti-rabbit IgG (Invitrogen, Carlsbad, CA, USA). Cell nuclei were labeled with Hoechst 33342 (Invitrogen). Samples were observed by Zeiss Axio Scope.A1 fluorescent microscope (HBO 100 lamp) (Carl Zeiss Microimaging GmbH, Göttingen, Germany). Images were analyzed with ZEN 2012 v.1.1.0.0. software (Carl Zeiss Microscopy GmbH, Göttingen, Germany), and for the NF-κB staining the ratio of nuclear and perinuclear fluorescence intensity was calculated. The specificity of immunostaining was checked by incubating the cells with the secondary antibody only, and no background staining was found.

### Investigation of TNF-α and everolimus-mediated transcriptional regulation of VCAM-1 and E-selectin by ChIP-seq

Processed ChIP-seq data were downloaded from the NCBI GEO depository (GEO accession number: GSE53998). Integrative Genomics Viewer (IGV2.3, Broad Institute) was used for data browsing [20] and creating representative snapshots. We reanalyzed the unstimulated and TNF-α-treated HUVEC cells-derived publicly available NF-κB transcription factor subunit p65, RNA Polymerase II (RNAPII), active histone mark H3K27Ac, and active transcription start site mark H3K4m3-specific ChIP-seq data sets. The method how enhancer RNAs (eRNA) were analyzed, was previously described by Brown *et al*. [21]. We wanted to identify TNF-α-activated transcription factor-bound enhancers in the neighboring genomic regions of VCAM-1 and E-selectin genes. HCAECs were then treated with TNF-α (100 ng/mL) with or without everolimus (0.5 μM) for 1 hour, and we then quantified the levels of two selected eRNAs (SELE_-11Kb and VCAM1_-10Kb) by RT-qPCR.

### MiRNA specific stem-loop RT-qPCR analysis

The expression of miRNAs was quantified in both types of EC cultures as well as in plasma samples by Universal ProbeLibrary (UPL)-probe based stem-loop RT-qPCR assay as we recently described [22]. The qPCR assays were designed by the software developed by Czimmerer *et al*. [23], and oligonucleotides used in this study are listed in S1 Table. This technique included two steps: 1) miRNAs (10 ng total RNA) were transcribed into cDNA via miRNA specific reverse transcription using miRNA-specific stem loop-RT primer (500 nM, IDT) and TaqMan^®^ MicroRNA^®^ Reverse Transcription Kit (ABI), and 2) miRNA quantification was performed by RT-qPCR using designed universal reverse primer (100 μM, Sigma-Aldrich), miRNA-specific forward primer (100 μM, IDT) and UPL probe #21 (10 μM, Roche Diagnostics) with Taq polimerase (5 U/μL, Thermo Scientific) and dNTPs (2.5 mM, Thermo Scientific). The reactions were incubated at 95°C for 1 min, followed by 40 cycles of 95°C for 15 sec and 60°C for 30 sec. All measurements were done in triplicates on a QuantStudio 12 K Flex qPCR instrument (ABI). Plasma miR-24 was found to have the most stable expression in patient samples, thus, this was applied for normalization of mature miRNAs as a reference gene throughout RT-qPCR analyses. For cell culture analyses, RNU-43 was used for normalization.

### Transfection of HCAECs with miR-181b mimic

The transfection of ECs with specific miR-181b mimic was performed based on the manufacturer’s instructions. Briefly, HCAECs were treated with TNF-α (100 ng/mL) for 1 hour in MesoEndo medium, and Opti-MEM I Reduced Serum Medium (Gibco) with 3% FBS, 100 U/ml Penicillin and 100 μg/ml Streptomycin was added to the cells for transfection. The overexpression of miR-181b was done using mirVana^®^ miR-181b mimic (25 pmol, Ambion, Austin, TX, USA) with Lipofectamine RNAiMAX^®^ Transfection Reagent (Invitrogen) for 24 hours at 37°C and 5% CO_2_. In parallel, negative control samples were treated with mirVana^®^ miRNA mimic negative control (NEG-01, 25 pmol, Ambion). After transfection, total RNA was extracted and this miRNA with SELE and VCAM1 mRNAs were quantified as described above.

### Quantification of pre-miRNAs and pri-miRNA levels in ECs exposed to TNF-α with or without everolimus treatment

We also studied the regulatory mechanisms how the expression of inflammation-specific miR-155 and E-selectin/VCAM-1 regulator miR-181b were modulated upon TNF-α stimulation of ECs in the presence or absence of everolimus. We analyzed the levels of both precursors of these miRNAs using RT-qPCR in HCAECs under the same experimental settings with TNF-α (100 ng/mL) and everolimus (0.5 μM) for 1 hour described above.

### Subjects

Subjects were previously characterized in our previous clinical study [6]. Briefly, 28 individuals were treated with BMS and 21 received everolimus-eluting stents. Six BMS subjects had ISR, while no complication was observed in the DES cohort in the first 6 months of stenting. These age- and gender-matched patient groups were comparable based on their baseline demographic and clinical parameters. The same regimen of aspirin and clopidogrel was administered in all patients until 1 month of follow-up period when these plasma samples were obtained. Thus, there was no clinical circumstances that might modify EC activation level and the RNA profile.

### Analysis of TNF-α levels in plasma samples

Plasma TNF-α concentrations were measured using commercially available ELISA kits (R&D Systems) according to the manufacturer’s instructions. Before performing the analysis, plasma samples were thawed and then centrifuged at 10,000 *g* for 1 min.

### Plasma samples for total RNA isolation

Venous blood samples collected into Vacutainer^®^ tubes containing 0.105 M sodium citrate (Becton Dickinson, San Jose, CA, USA) were subsequently centrifuged at 170 *g* for 15 min at room temperature (RT) to obtain platelet-rich plasma (PRP) samples, which were further centrifuged at 1500 *g* for 15 min to obtain platelet-poor plasma (PPP). These samples had been stored at -70°C before total RNA was extracted. Prior to RNA isolation, PPP samples were thawed once and 750 μL TRI reagent (MRC) was added into 250 μL PPP, and total RNA was isolated according to the manufacturer’s recommendations.

### Ethics statement

This study was approved by the Regional Ethics Committee of the University of Debrecen (permit number: 4102/2014) in accordance with the Declaration of Helsinki. All participants gave their written informed consent.

### Statistical analyses

Data are expressed in mean ± standard error of the mean (SEM). Comparison of multiple groups was performed using ANOVA or Kruskal-Wallis with *post hoc* test, while *t*-test was performed to compare two groups of data. The Kolmogorov-Smirnov test was used for the evaluation of the normality of the data. Pearson’s correlation coefficient (r) was used to explore relationship between the levels of soluble adhesive receptors and circulating miR-181b. P≤0.05 probability level was regarded as statistically significant. Analyses were performed using GraphPad Prism, version 6.01 (GraphPad Software, La Jolla, CA, USA).

## Results

### Elevated E-selectin and VCAM-1 mRNA levels induced by TNF-α were downregulated by everolimus in ECs *in vitro*

**Fig 1:**
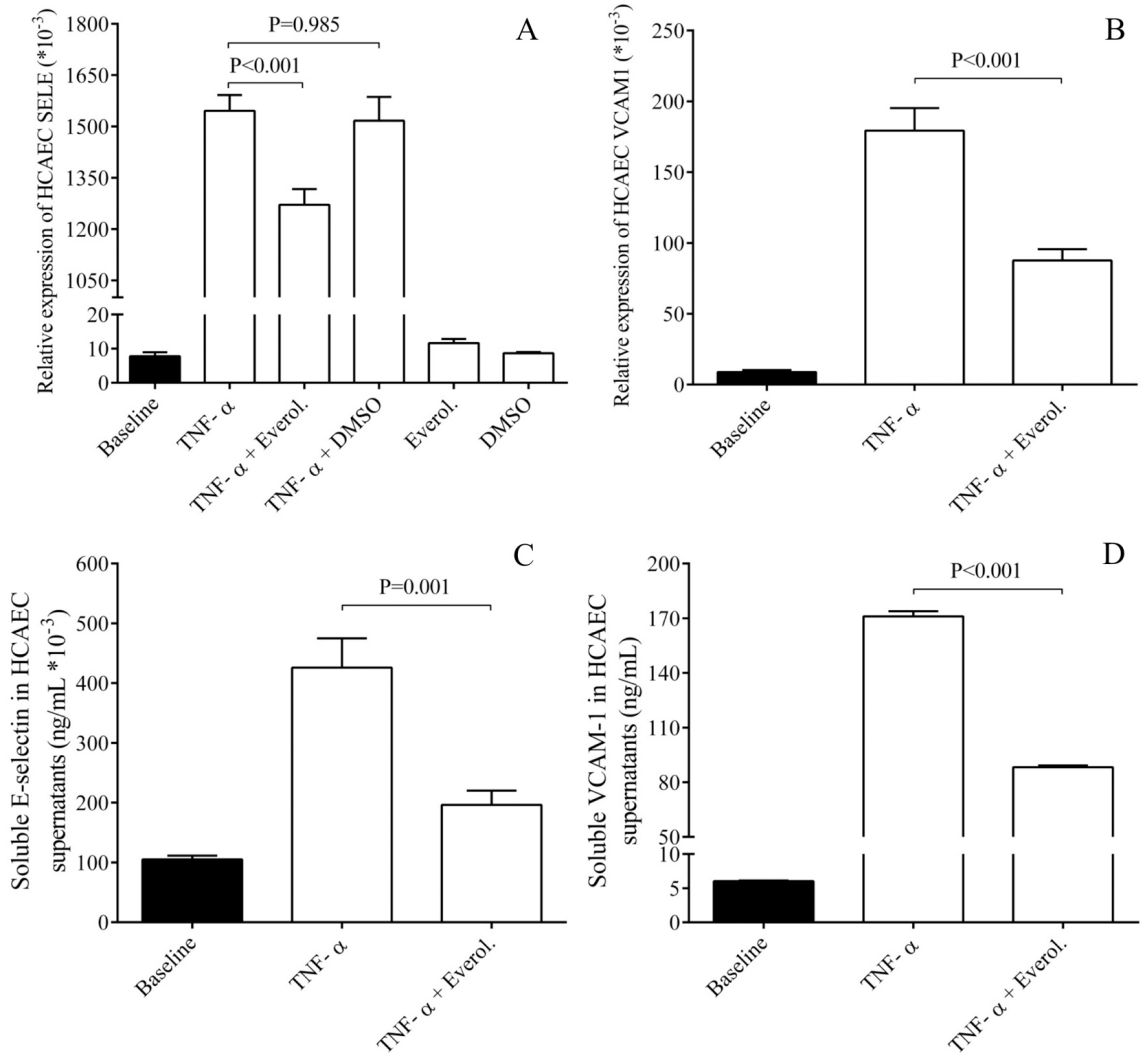
Analysis of E-selectin and VCAM-1 expression at mRNA and protein levels in HCAECs after TNF-α stimulation. HCAEC cells were treated with recombinant TNF-α (100 ng/mL) for 1–24 hours to generate cellular inflammatory conditions. Elevated E-selectin (SELE) (A) and VCAM-1 mRNA levels (B) induced by TNF-α already after 1 hour were downregulated by everolimus in HCAECs *in vitro*. In parallel, soluble E-selectin (C) and VCAM-1 concentrations (D) were measured by ELISA in the supernatants of ECs and were also significantly decreased after 4 and 24 hours, respectively. Mean ± SEM, n = 4-8/group.

There was a higher EC activation level in BMS patients with ISR compared to DES subjects without any complication [6]. Thus, we first investigated whether elevated expression of EC activation dependent adhesion molecules E-selectin and VCAM-1 could be observed in EC cultures under inflammatory conditions with or without everolimus *in vitro*. Our aim was to study the potential regulatory mechanisms of EC activation that might be caused by distinct coronary stents. E-selectin and VCAM-1 mRNA levels were analyzed in HCAECs and HUVECs after treatment with TNF-α in the presence or absence of everolimus. TNF-α stimulation resulted in a robust elevation in both mRNA levels compared to baseline sample. In contrast, everolimus in the presence of TNF-α significantly, however not completely lowered these mRNA levels in HCAECs (P<0.001) (Fig 1A and 1B) and in HUVECs (P<0.001) as well (S1A and S1B Fig). No alteration in these mRNA levels was found by everolimus alone or by vehicle (DMSO) with TNF-α vs. untreated baseline samples.

To provide further evidence about the effect of everolimus on the expression of these adhesive receptors, their concentrations were also determined by ELISA in the supernatants of HCAEC samples after treatment by TNF-α in the absence and presence of everolimus. Inflammation-raised E-selectin and VCAM-1 concentrations were significantly decreased by everolimus (P = 0.001, P<0.001, respectively) (Fig 1C and 1D) in agreement with their altered mRNA levels in ECs above. Hence, these *in vitro* results provide some explanation about the lower level of EC activation with less E-selectin/VCAM-1 in DES individuals.

### Everolimus lowered EC inflammation via lowered IL-1β and IL-6 expression in ECs

**Fig 2:**
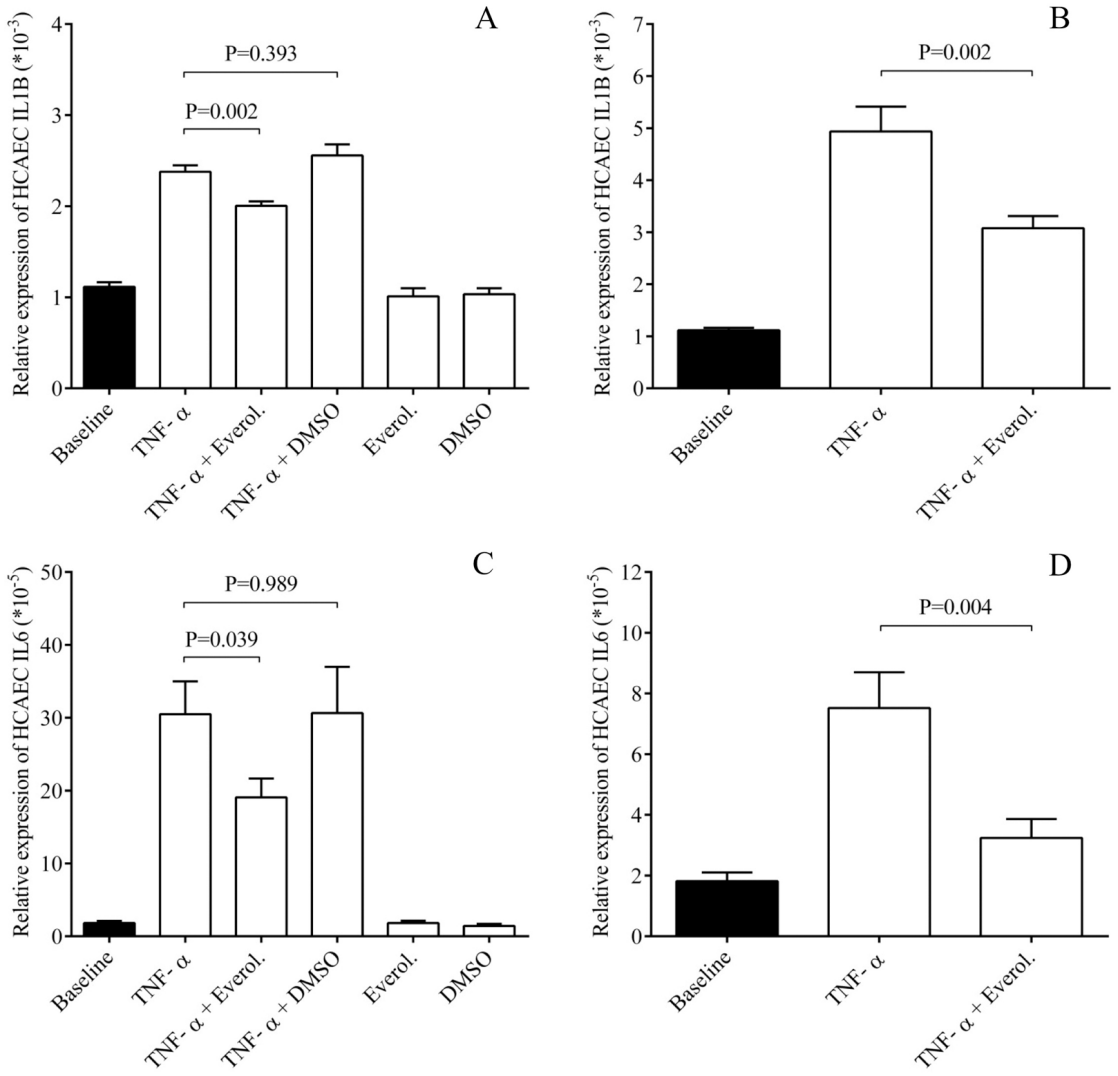
Everolimus decreased EC inflammation via lowered IL-1β and IL-6 expression in ECs. HCAECs were treated with TNF-α (100 ng/mL) with or without everolimus (0.5 μM) for 1 and 4 hours, and then IL-1β and IL-6 mRNA levels were quantified by RT-qPCR. This effect was observed already after 1 hour (A, C), while was more pronounced after 4 hours (B, D). Mean ± SEM, n = 4-8/group.

To investigate if everolimus downregulates adhesive molecule expression via modulating a global inflammatory response in ECs, HCAECs were treated with TNF-α that caused a robust elevation in IL-1β and IL-6 mRNA levels already by 1 hour (Fig 2A and 2C), and further increased by 4 hours (Fig 2B and 2D). In contrast, everolimus moderately but significantly lowered IL-1β (P = 0.002) and IL-6 mRNAs (P = 0.039) already after 1 hour, which were more obvious after 4 hours of treatment (P = 0.002, P = 0.004, respectively). Based on these results, TNF-α-induced EC inflammation could be interrupted by everolimus.

### Effect of everolimus on TNF-α-induced NF-κB nuclear translocation in HCAECs

**Fig 3:**
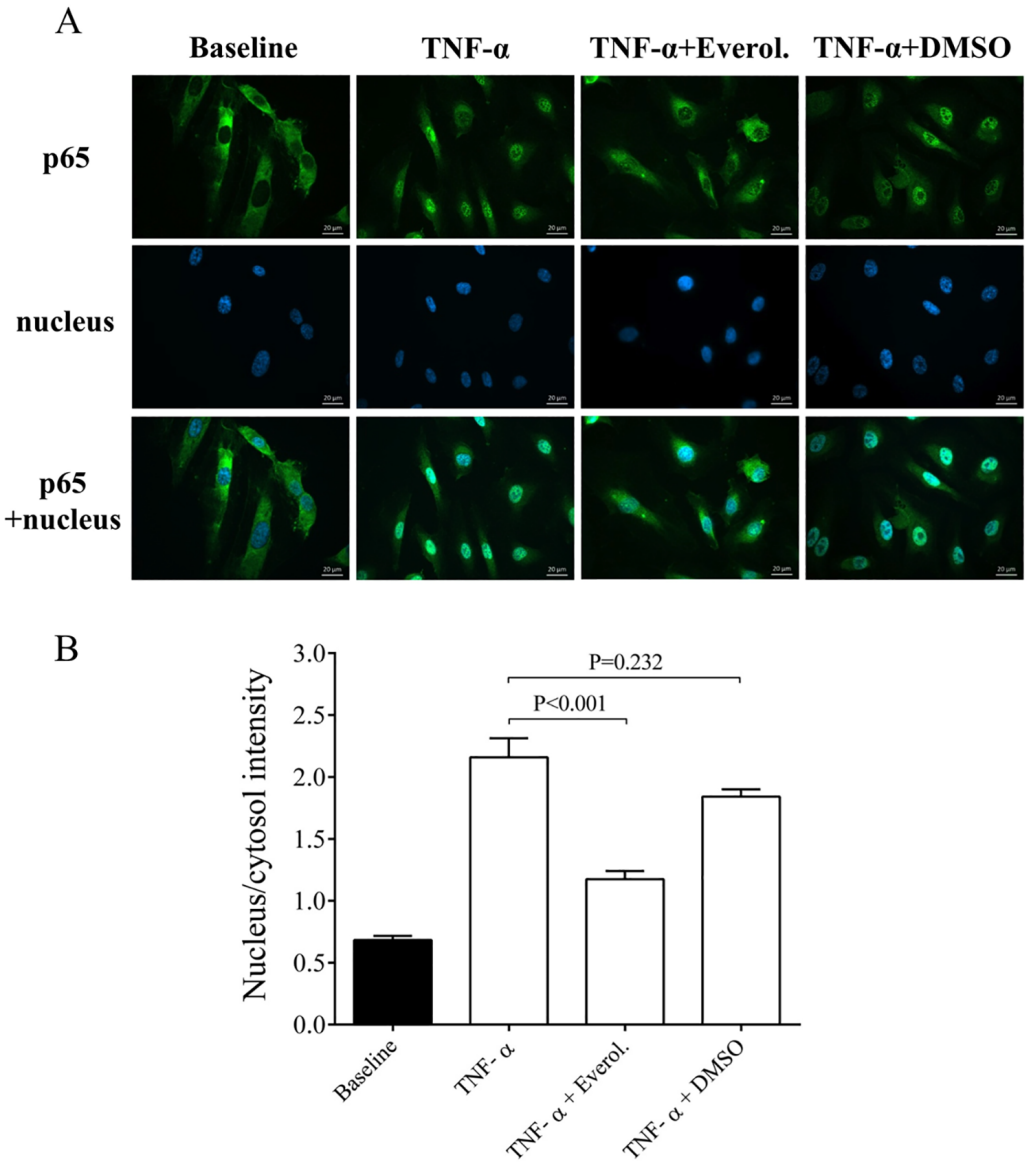
Immunohistochemical staining and analysis of NF-κB activation in TNF-α and everolimus treated endothelial cells. HCAEC cells were treated for 1 hour with MesoEndo Medium (Baseline), 100 ng/ml TNF-α with or without 0.5 μM everolimus or cytokine with the solvent of everolimus (TNF-α+DMSO). Nuclear localization of the NF-κB p65 subunit was monitored by immunostaining. Green: p65 staining; blue: cell nuclei. Scale bar: 20 μm (A). Ratio of the fluorescence intensity of the NF-κB immunostaining in cell nuclei and cytosol was analyzed (B). Mean ± SEM, n = 6-8/group.

Translocation of the p65 NF-κB subunit into the cell nuclei reliably evaluates the degree of an inflammatory reaction at cellular level [19]. To further analyze the direct effect of everolimus in inflammation-stimulated ECs, early NF-κB p65 nuclear translocation was studied in HCAEC cultures after stimulation with cell culture medium, or TNF-α (100 ng/mL) in the absence or presence of everolimus (0.5 μM) or with its solvent for 1 hour. The nucleus/cytosol intensity of p65 staining was studied in these cells. We found that compared to TNF-α stimulated cells, everolimus treatment in the presence of TNF-α significantly decreased the p65 staining in the cell nucleus (P<0.001). In contrast, TNF-α+DMSO sample did not show alteration in p65 staining (Fig 3A and 3B). Accordingly, everolimus was shown to directly influence EC activation via interrupting NF-κB pathway.

### Transcriptional regulation of E-selectin and VCAM-1 expression contributed to everolimus effects

**Fig 4:**
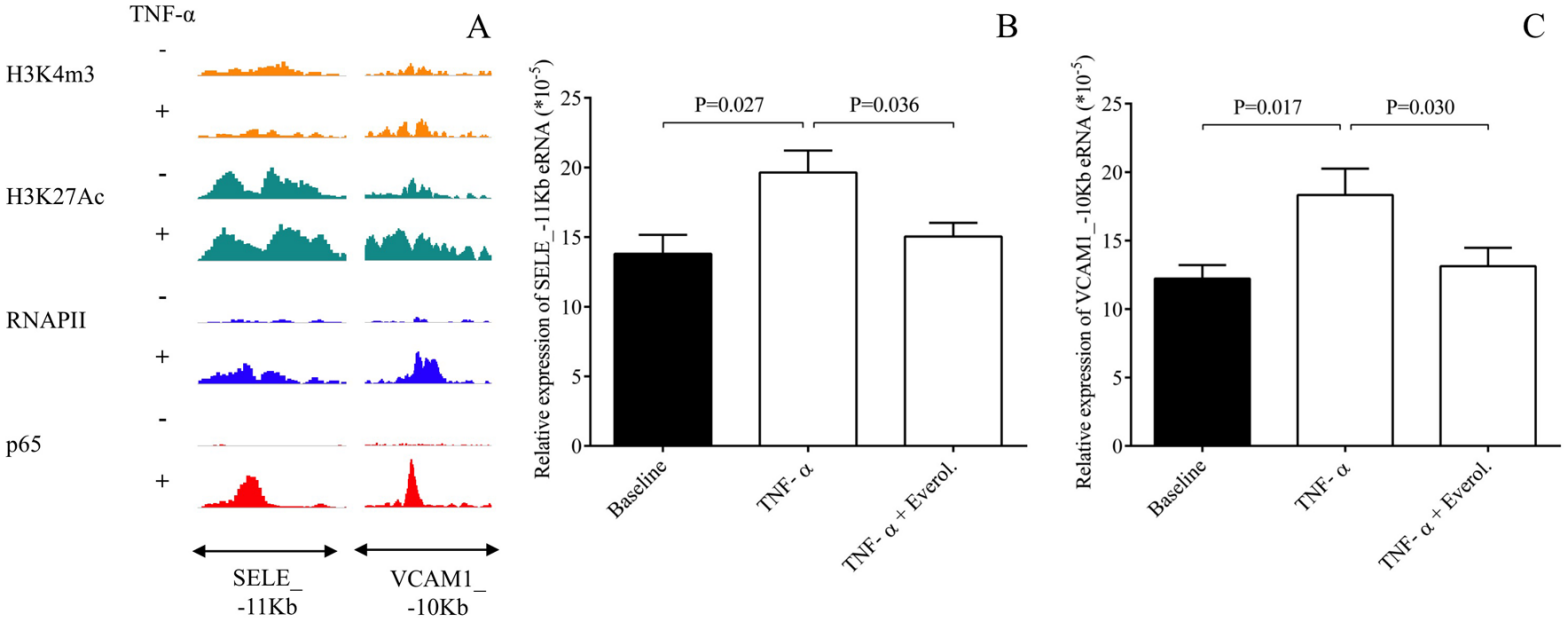
Analysis of transcriptional regulation of SELE and VCAM-1 genes in unstimulated and TNF-α-treated HUVECs using publicly available ChIP-seq data sets and the measurement of eRNA expression at two selected enhancers including SELE_-11Kb and VCAM1_-10Kb in HCAECs. The p65, RNAPII, H3K27Ac and H3K4m3-specific ChIP-seq signals at the selected VCAM1 and SELE-associated enhancers were visualized by the Integrative Genomics Viewer (A). Accordingly, HCAECs were then treated with TNF-α (100 ng/mL) with or without everolimus (0.5 μM) for 1 hour, and one-one eRNA expression at SELE_-11Kb (B) and VCAM1_-10Kb eRNAs (C) were quantified by RT-qPCR. TNF-α-augmented expression of eRNAs was significantly reduced by everolimus treatment. Mean ± SEM, n = 4-8/group.

The expression of E-selectin and VCAM-1 are transcriptionally regulated in ECs in an inflammatory signaling dependent manner, induced by e.g. lipopolysaccharides (LPS) [24]. For further investigation of potential TNF-α and everolimus-modulated transcriptional regulation of E-selectin and VCAM-1 expression, we here re-analyzed the unstimulated and TNF-α-treated HUVEC-derived publicly available NF-κB transcription factor subunit p65, RNA Polymerase II (RNAPII), active histone mark H3K27Ac, and active transcription start site mark H3K4m3-specific ChIP-seq data sets [20]. As we expected, TNF-α-induced RNAPII binding was observed at both SELE and VCAM1 gene bodies (S2A and S2B Fig). In addition, two enhancers were identified in the neighboring genomic regions of both genes associating with TNFα-induced p65 and RNAPII binding (Fig 4A, S2A and S2B Fig). Recent studies showed that eRNA expression is a good marker of enhancer activity and is regulated in similar manner as the neighboring genes in many cell types by different signals [20, 25–27]. Therefore, we measured the eRNA expression at one-one selected TNF-α-activated p65 transcription factor-bound enhancers in the neighboring genomic regions of both genes including SELE_-11Kb and VCAM1_-10Kb in unstimulated, TNF-α as well as TNF-α and everolimus-treated HCAECs using RT-qPCR method. TNF-α induced eRNA expression at both enhancers compared to the baseline sample. However, the TNF-α-augmented eRNA expression was significantly reduced by everolimus treatment (P = 0.036, P = 0.030, respectively) (Fig 4B and 4C). Overall, we suppose that everolimus inhibits EC activation via altering the TNF-α-induced transcription of EC activation-related genes, such as SELE and VCAM1.

### TNF-α induced EC inflammation was associated with decreased miR-181b

**Fig 5:**
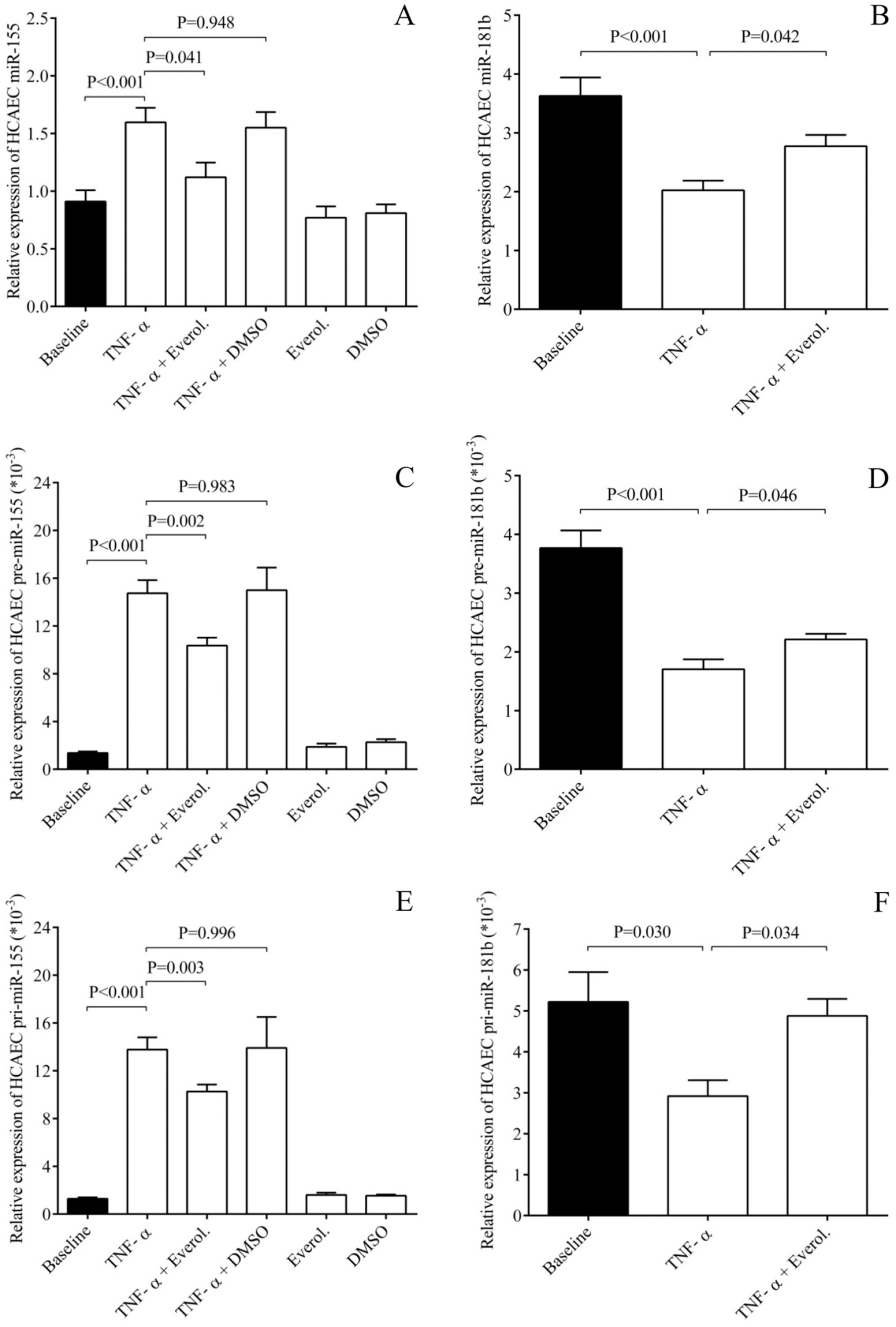
Quantification of TNF-α induced miR-155 and miR-181b levels with the analysis of their precursors in the presence of everolimus upon EC inflammation *in vitro*. HCAECs were treated by recombinant TNF-α for 1–4 hours to analyze miR-181b expression along with the inflammation-specific miR-155. First, miR-155 was elevated by TNF-α compared to untreated sample, however, everolimus caused significantly decreased miR-155 levels (A), while miR-181b was downregulated by the inflammatory stimulus and the treatment with everolimus restored their expression in both EC cultures (B). Levels of pre- and pri-miRNA were altered in the same manner as seen in mature miR-155 (C, E) and miR-181b (D, F), respectively. Mean ± SEM, n = 4-8/group.

Although E-selectin and VCAM-1 expression were found to be highly regulated at transcriptional level in this experimental system, we sought to study the role of post-transcriptional regulator of these receptors upon EC inflammation. Since miR-181b modulated VCAM-1 and E-selectin expression in HUVECs among *in vitro* conditions [17], we here analyzed the levels of this miRNA in TNF-α-stimulated ECs with or without everolimus as their potential key effector. Both HCAECs and HUVECs were treated by recombinant TNF-α for 1–4 hours to analyze miR-181b expression along with inflammation-specific miRNAs [25]. As expected, miR-155 and miR-146a as well as the biomarker of EC dysfunction miR-185 [28, 29] were elevated by TNF-α compared to untreated sample in both EC cultures (Fig 5A and S3A and S3B Fig). However, everolimus caused significantly decreased miR-155 and miR-146a levels, with lower miR-185 expression (data not shown). Importantly, the level of miR-181b was downregulated by the inflammatory stimulus (P<0.001) and the treatment with everolimus restored their expression in both EC cultures (P = 0.042, P = 0.049) (Fig 5B and S3C Fig). As control, we checked that the vehicle (DMSO) with TNF-α and everolimus alone were unable to alter these miRNAs.

### Precursors of miRNAs were also altered by everolimus in ECs

We subsequently studied whether altered levels of these mature miRNAs above were due to their abnormal transcriptional regulation. Therefore, the levels of pre- and pri-miR-155, and both precursors of miR-181b were quantified by RT-qPCR in HCAECs stimulated with TNF-α with or without everolimus (Fig 5C–5F). We found that the levels of these miRNA precursors were altered in the same manner as seen in mature miRNAs. These findings suggest that miR-155 (C, E) and miR-181b (D, F) expression were modulated at transcription level by TNF-α stimulation and everolimus in ECs.

### Endothelial cell miR-181b regulates the SELE and VCAM1 expression

**Fig 6:**
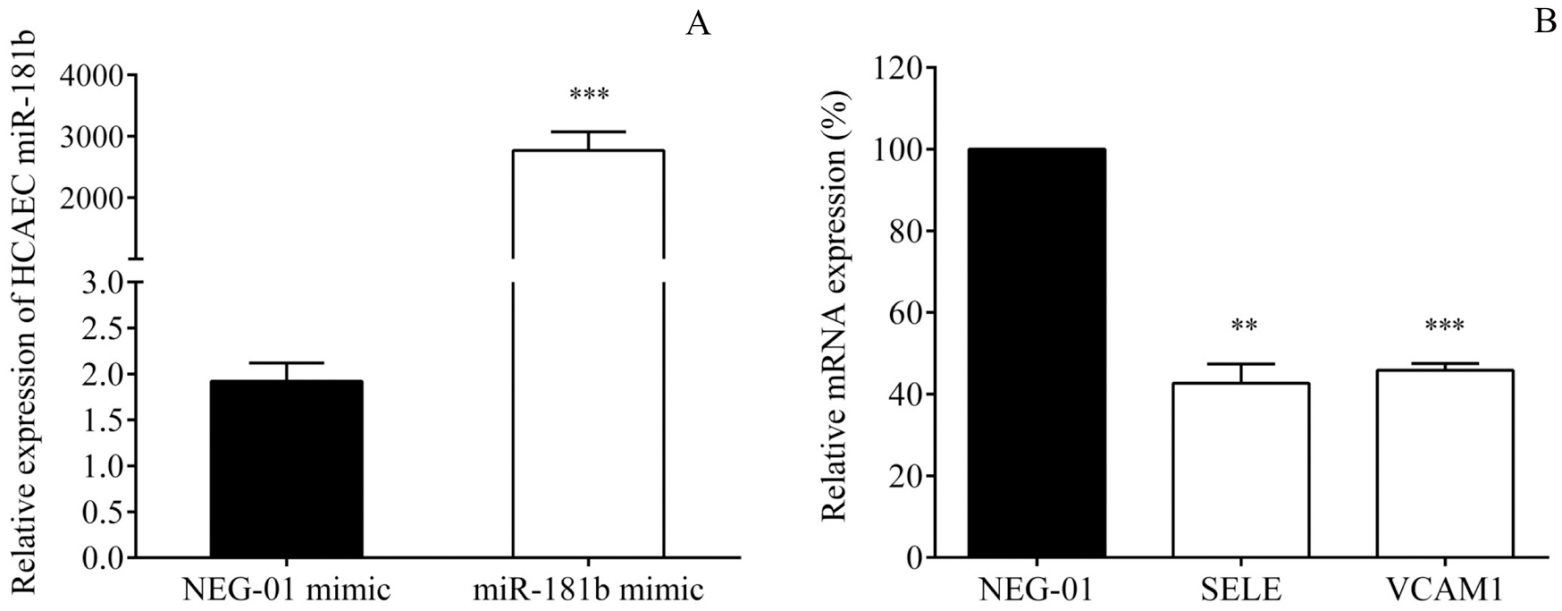
Overexpression of miR-181b altered the levels of SELE and VCAM1 mRNA in HCAECs. The direct association between miR-181b and SELE/VCAM1 was investigated in HCAECs after stimulation with TNF-α by using transfection of mirVana^®^ miR-181b mimics (25 pmol) with Lipofectamine RNAiMAX^®^ Transfection Reagent for 24 hours. In parallel, negative control samples were treated with mirVana^®^ miRNA mimic negative control (NEG-01, 25 pmol). After transfection, miR-181b with SELE and VCAM1 mRNAs were quantified by RT-qPCR. Highly increased miR-181b levels (A) resulted in significantly lowered SELE and VCAM1 mRNAs compared to NEG-01 control samples (100%, B). **P = 0.006, ***P<0.001 based on t-test. Mean ± SEM, n = 4/group.

Despite some former available data revealed in HUVECs [17], we wanted to confirm the relationship between miR-181b and SELE and VCAM1 in HCAECs stimulated with TNF-α by using transfection of specific miR-181b mimic. The overexpression of miR-181b was produced by its specific mimic (Fig 6A), however, the levels of other miRNAs, e.g. miR-155, were not affected (data not shown). As a consequence, SELE (P = 0.006) and VCAM1 mRNA levels (P<0.001) were significantly decreased in the coronary endothelial cells versus control samples transfected with the NEG-01 control mimic (Fig 6B). Based on these results, we confirmed that miR-181b targets E-selectin and VCAM-1 in HCAECs.

### Impaired plasma miR-181b correlates with increased plasma levels of related soluble E-selectin and VCAM-1 concentrations

BMS patients with ISR showed higher level of EC activation in contrast to those who received everolimus eluting coronary stent [6]. Former data on soluble E-selectin and VCAM-1 were re-analyzed for this study and depicted in S4A and S4B Fig. There was significantly elevated soluble E-selectin concentrations in BMS subjects with ISR (P = 0.032) (S4A Fig), while VCAM-1 levels were markedly higher (P = 0.160) in comparison with DES cohort without any clinical complication (S4B Fig). Furthermore, to gain more direct evidence about the distinct effect of BMS and DES on vascular inflammation, we determined the level of TNF-α levels in the plasma samples of our stented patients by ELISA. We found that TNF-α levels were significantly lower in those subjects with DES compared to individuals with BMS having ISR (P = 0.049) (S5 Fig). Alterations in these receptor expressions and the level of pro-inflammatory cytokine indicated more EC activation when ISR was developed by BMS without the protective effect of everolimus.

**Fig 7:**
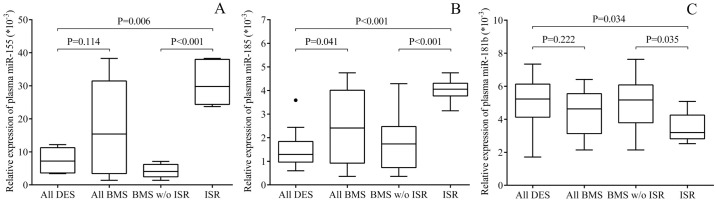
Quantification of circulating miR-155, miR-185 and miR-181b by RT-qPCR in plasma samples of BMS and DES patients. After total RNA isolation, the expression of circulating miRNAs was quantified in plasma samples by UPL-probe based stem-loop RT-qPCR assay. Plasma miR-155 (A) and miR-185 (B) were significantly upregulated in BMS patients with ISR compared to BMS and DES subjects without any complications, while miR-181b levels (C) were lower in those with ISR versus others without complication. (All DES: n = 21, all BMS: n = 28, ISR: n = 6).

The circulating level of miR-155, miR-185 and miR-181b were quantified by RT-qPCR in the plasma samples of the entire patient population. We sought to investigate if these plasma miRNAs were changed in the same way as seen in ECs *in vitro*, and to support our findings about abnormal concentrations of soluble adhesive molecules and EC inflammation/dysfunction in case of ISR. First, plasma miR-155 (P = 0.006) and miR-185 (P<0.001) were significantly upregulated in BMS patients with ISR compared to BMS and DES subjects without any complication (Fig 7A and 7B). These results revealed the presence of EC inflammation and dysfunction with typical miRNA alterations in those with ISR. Of note, we found some difference between distinct types of stent showing lower inflammation-specific miRNAs in DES subjects. Furthermore, the levels of VCAM-1 and E-selectin repressor miR-181b were significantly lower in BMS+ISR as compared to other BMS (P = 0.035) or DES implantation without complication (P = 0.034) (Fig 7C). MiR-34a and miR-126 being considered as key miRNAs in vascular inflammation [16,30], were also measured as ‘control miRNAs’ in these plasma specimens and showed lower levels in ISR versus DES individuals (P<0.001, P = 0.036, respectively) (S6A and S6B Fig), which were comparable to former results by others [16, 30]. These data supported the validity of our patient samples representing pathological vascular conditions after coronary stenting. Finally, correlation tests were performed to study the relationship between plasma VCAM-1 or E-selectin concentrations and miR-181b levels in the pooled patient samples. In accordance with the former data of Sun *et al*. [17] and the effect of miR-181b on SELE/VCAM1 we detected (Fig 6), plasma miR-181b expression showed a significant negative correlation with VCAM-1 and E-selectin concentrations (r = -0.441, P = 0.019; r = -0.375, P = 0.049, respectively) (data not shown).

**Fig 8:**
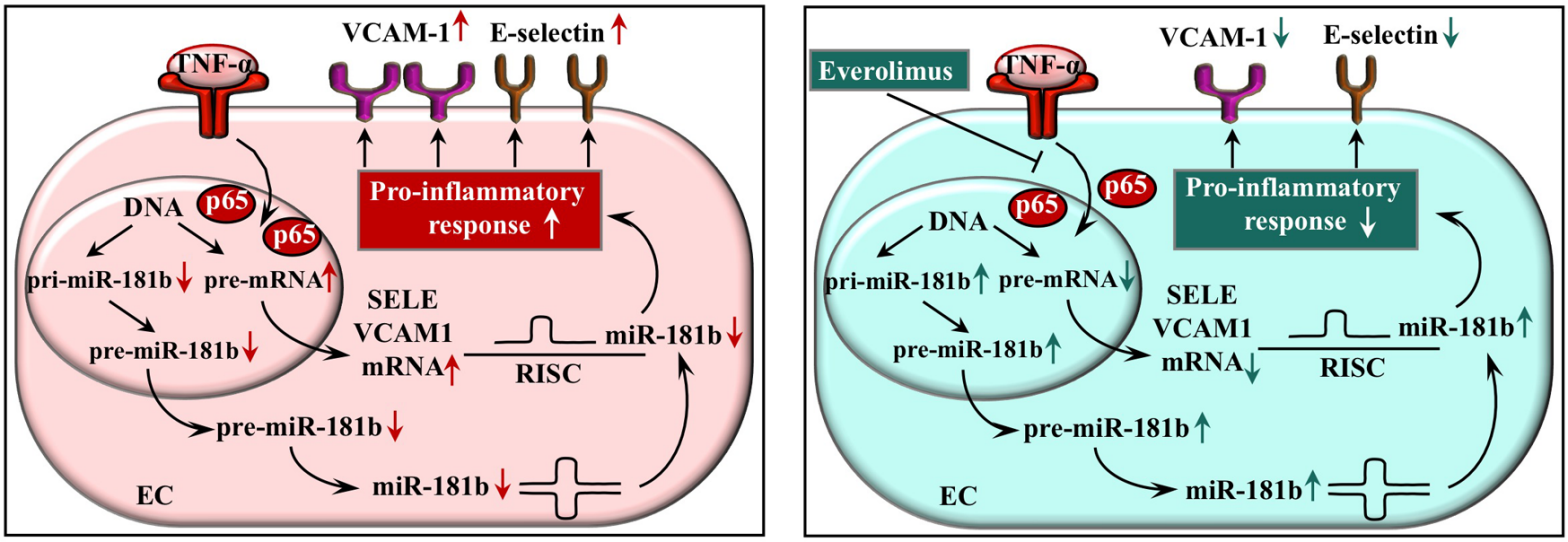
Schematic figure about the model to demonstrate the regulatory mechanisms of everolimus on E-selectin (SELE) and VCAM-1 expression. Everolimus decreases EC activation via suppressing the NF-κB pathway with decreased p65 translocation into cell nuclei causing the modulation of E-selectin and VCAM-1 expression as well as miR-181b level at transcriptional and post-transcriptional level, respectively. EC: endothelial cell, TNF-α: tumor necrosis factor alpha, SELE: E-selectin, VCAM-1: vascular cell adhesion molecule-1, RISC: RNA-induced silencing complex.

Based on all these data, we propose a model where everolimus acts as a negative regulator of EC activation via inhibiting NF-κB p65 subunit translocation resulting in altered E-selectin and VCAM-1 mRNA levels with their post-transcriptional repressor miR-181b at transcription level (Fig 8). These cellular events may occur in the early phase of DES implantation showing a beneficial protective effect of everolimus as compared to bare-metal stenting without any drug elution.

## Discussion

Despite recent development in coronary stenting, ISR associated with VSMC proliferation and EC injury still represent a major clinical issue in those who undergo such intervention [1], and is often associated with enhanced platelet activation with increased amount of microparticles as well [31]. The introduction of DES coated with different drugs, e.g. mTOR inhibitors, has substantially reduced the risk for complications, such as ISR in contrast to BMS in the early phase of stenting [1, 2]. Sirolimus during angioplasty prevented VSMC proliferation in an animal model [32], and antagonized VCAM-1 levels induced by TNF-α in cultured HUVECs via inhibiting mTORC2 activity and potentiated ERK1/2 [11]. However, no studies have investigated the potential transcriptional and post-transcriptional regulatory mechanisms by which everolimus can decrease EC activation. Of note, these drugs may result in late in-stent thrombosis after 1–2 years of intervention [1, 33], which may be due to the upregulation of plasminogen activation inhitor-1 by ECs [34].

Recently, higher level of EC activation was described with elevated soluble E-selectin and VCAM-1 levels after BMS intervention versus in DES individuals [4–6, 8]. Moreover, TNF-α levels were significantly lower in our subjects with DES compared to individuals with BMS having ISR (S5 Fig). These results were in agreement with the paper of McNair *et al*. [8]. Adhesion receptors are expressed on activated ECs stimulated by TNF-α and other inflammatory cytokines, and then involved in leukocyte attachment to ECs [7]. DES displays a beneficial effect to prevent or at least slow down the development of EC activation/dysfunction leading to ISR via locally maintained everolimus concentration of cc. 0.5 μM in stented vessels [35]. This anti-proliferative drug has also showed a substantial anti-inflammatory effect in neutrophils reducing the release of IL-8 and decreasing TNF-α-induced adhesion of neutrophils to ECs [10]. However, no data are available by which mechanisms everolimus can lower EC activation, such as after DES stenting. Hence, in this study, we have systematically investigated the potential transcriptional and post-transcriptional regulatory mechanisms of everolimus.

We first investigated the contribution of transcriptional regulatory mechanisms to the TNF-α and everolimus-dependent regulation of EC activation-linked genes, such as E-selectin and VCAM-1. Cultured HCAECs and HUVECs were challenged with 100 ng/ml concentration of recombinant TNF-α—similarly to Palmieri *et al*. [36]—to investigate vascular inflammation in the presence or absence of everolimus after stenting. Initially, we applied HCAECs as reliable cell culture for the investigation of RNA levels in arterial endothelium [37], while results were also confirmed in HUVECs, which is a fundamental cell culture model to analyze endothelial responses to distinct challenges [18, 38].

To analyze the direct effect of everolimus on EC function upon inflammation, we added this drug to the medium of EC cultures in the presence of TNF-α. Everolimus substantially decreased the NF-κB pathway via prevention of p65 translocation into cell nuclei and partially or completely inhibited the TNF-α-dependent effects on E-selectin and VCAM-1 transcription indicating that it effectively antagonized EC activation. Concentrations of VCAM-1 and E-selectin showed similar alterations in the supernatants of ECs *in vitro* upon inflammation and everolimus treatment. We also assessed very low (0.05 μM) and high (5 μM) concentration of everolimus during the setting of these experiments. No difference in RNA levels was observed at low concentration, however, its high concentration resulted in the apoptosis of ECs (data not shown). In agreement with our results, LPS-induced EC activation was also downregulated by rapamycin via mTOR/NF-κB pathway in HUVECs [38]. Our current RT-qPCR-based measurements and the re-analysis of publicly available ChIP-seq data sets of HUVECs showed that these genes and SELE_-11Kb and VCAM1_-10Kb eRNAs are activated in ECs by TNF-α confirming their TNF-α-dependent transcriptional regulation. Importantly, everolimus decreased the levels of these eRNAs. Others recently described the NF-κB binding site in the -1643 and -1652 regions of miR-99a promoter by ChIP assay, and this miRNA modulated EC inflammation in HUVECs [38].

MiRNAs play a major role in the post-transcriptional regulation of EC activation-dependent events in coronary artery disease [12, 39]. MiR-223 and miR-141 suppressed ICAM-1 expression in EC cultures *in vitro* [40, 41], while NF-κB mediated inflammation was regulated by elevated expression of miR-146a and miR-155 [25, 39]. Moreover, PCI-induced plaque rupture was associated with increased miR-155 levels [42]. A number of miRNAs are involved in the development of EC dysfunction, such as upregulated miR-185 in response to high glucose milieu [28], miR-99a in LPS-stimulated [38], and miR-149 in TNF-α-induced EC dysfunction through p38MAPK [36]. These previous data revealed a functional role of these miRNAs in the regulation of various pathological cellular events during vascular inflammation at post-transcriptional level. In our study, TNF-α enhanced miR-146a, miR-155 and miR-185 expression in both EC cultures indicating the cellular inflammatory response and dysfunction as seen earlier [41]. Importantly, we also observed that miR-181b targeted SELE and VCAM1 mRNAs, and TNF-α transcriptionally repressed miR-181b expression suggesting that TNF-α may enhance E-selectin and VCAM-1 at post-transcriptional level. MiR-181b inhibits importin-α3 expression and NF-κB-responsive VCAM-1 and SELE genes [17]. In other experiments, TNF-α treatment in HUVECs resulted in decreased miR-141 levels causing enhanced ICAM-1 expression [41], while miR-149 was also decreased due to the same response affecting IL-6 and metalloproteinase-9 expression in EC cultures [38]. Overall, these miRNAs do not only represent a new layer of regulation but may act as new biomarkers in cardiovascular diseases [15].

Here, Pearson’s correlation tests demonstrated a significant reverse correlation between plasma miR-181b levels and plasma VCAM-1 and E-selectin concentrations in our stented patient cohort supporting the relationship between the levels of this post-transcriptional regulator and the expression of these adhesive proteins. Based on our data, decreased plasma miR-181b level may be useful to indicate stent-induced EC activation with enhanced VCAM-1 and E-selectin expression. In apolipoprotein E-deficient/NF-κB-luciferase transgenic mice miR-181b significantly inhibited atherosclerotic lesion formation, pro-inflammatory gene expression and the influx of lesional macrophages and CD4+ T cells in the vessel wall suggesting the central role of this miRNA in vascular inflammation during atherosclerosis [43].

Our study has some limitations. The number of patients with ISR was relatively small in our former clinical study [6], which was obviously due to the fact that it was a single center study with a limited number of eligible patients per year. Thus, more data are needed to observe the relationship of miR-181b level with soluble E-selectin/VCAM-1 concentrations as a potential biomarker of EC activation and ISR.

We here described for the first time the transcriptional regulatory effects of everolimus on EC inflammation in details. These transcriptional (p65-bound eRNAs SELE_-11Kb and VCAM1_-10Kb) and post-transcriptional regulators (miR-181b) may represent potential therapeutic targets upon EC dysfunction. However, further studies are required to prove the functional role of these regulators *in vivo*. Similarly, neo-intimal formation and ISR development were effectively modulated by anti-miR-21 [44], or by the overexpression of miR-23b [45] based on the results of animal models. These data propagate to introduce novel ‘drug’-eluting stents for patients in the near future. As such, a stent system that eluted miR-126 exhibited significant inhibition of neointimal formation in a rabbit model of restenosis [46].

## Conclusions

We provide some pieces of evidence that everolimus acts as a negative regulator of EC activation via suppressed NF-κB pathway with lower p65 translocation into cell nuclei, the modulation of the expression of SELE and VCAM-1 and their post-transcriptional repressor miR-181b at transcription level. These data may explain how the level of EC activation can be lowered by everolimus when DES is used for coronary intervention in contrast to BMS implantation.

## Supporting information

S1 Table Sequences of primers for the analysis of mature and precursor miRNAs as well as mRNAs and eRNAs. (XLSX)

S1 Fig Measurement of E-selectin and VCAM-1 mRNAs in HUVECs after treatment with TNF-α. HUVECs were treated with recombinant TNF-α (100 ng/mL) for 4 hours to generate cellular inflammatory conditions. Elevated E-selectin (SELE) (A) and VCAM-1 mRNA levels (B) induced by TNF-α were downregulated by everolimus in HUVECs *in vitro*. Mean ± SEM, n = 4-8/group.
(TIF)

S2 Fig Analysis of the p65, RNAPII, H3K27Ac and H3K4m3-specific ChIP-seq signals at the genomic loci of VCAM1 and SELE visualized by the Integrative Genomics Viewer. The unstimulated and TNF-α-treated HUVEC-derived publicly available NF-κB transcription factor subunit p65, RNA Polymerase II (RNAPII), active histone mark H3K27Ac, and active transcription start site mark H3K4m3-specific ChIP-seq data sets were reanalyzed. The identified TNF-α-activated p65-bound enhancers in the neighboring genomic regions of SELE (A) and VCAM1 (B) genes were indicated by red arrows.
(TIF)

S3 Fig Quantification of TNF-α induced miR-146a, miR-155 and miR-181b levels upon inflammation in HUVECs *in vitro*. HUVECs were treated with TNF-α (100 ng/mL) with or without everolimus (0.5 μM) for 1 and 4 hours, and then these miRNA levels were quantified by RT-qPCR. Everolimus caused significantly decreased miR-146a (A) and miR-155 levels (B). The level of miR-181b (C) was downregulated by the inflammatory stimulus and the treatment with everolimus restored their expression in these EC cultures as well. Mean ± SEM, n = 4-8/group.
(TIF)

S4 Fig Re-analysis of soluble E-selectin and VCAM-concentrations in plasma samples of BMS and DES subjects with or without ISR. There were significantly higher E-selectin (A) and markedly elevated VCAM-1 levels in those who had ISR (n = 6) compared to other BMS (n = 22) and DES (n = 21) individuals [6]. Mean ± SEM.
(TIF)

S5 Fig Investigation of TNF-α concentrations in plasma samples of patients underwent BMS or DES. There were significantly higher plasma levels of TNF-α in those subjects who received BMS and showed ISR (n = 6) compared to individuals with DES (n = 21). Mean ± SEM.
(TIF)

S6 Fig Analysis of plasma miR-34a and miR-126 expression in the presence or absence of ISR in BMS and DES patients. After total RNA isolation, the expression of circulating miRNAs was quantified in plasma samples by UPL-probe based stem-loop RT-qPCR assay. These miRNAs were significantly lower in those with ISR compared to BMS and DES subjects without such complication. Mean ± SEM. (All DES: n = 21, all BMS: n = 28, ISR: n = 6).
(TIF)

## Abbreviations

AMI: acute myocardial infarction
BMS: bare metal stent
CAD: coronary artery disease
cDNA: complementary DNA
ChIP-Seq: chromatin immunoprecipitation sequencing
CXCL12: C-X-C motif chemokine 12
DES: drug-eluting stent
DM: diabetes mellitus
DMSO: dimethyl sulfoxide
EC: endothelial cell
ELISA: enzyme-linked immunosorbent assay
ERK1/2: extracellular signal-regulated protein kinases 1 and 2
eRNA: enhancer RNA
FBS: Fetal Bovine Serum
H3K27AC: acetylation lysine 27 on the histon H3 protein subunit
H3K4ME3: trimethylation of lysine 4 on the histone H3 protein subunit
HCAEC: human coronary artery endothelial cell
HUVEC: human umbilical vein endothelial cell
ICAM-1: intercellular adhesion molecule-1
IL-1β: interleukin-1 beta
IL-6: interleukin 6
ISR: in-stent restenosis
miRNA: microRNA
mRNA: messenger RNA
mTOR: the mammalian target of rapamycin
mTORC2: mTOR complex 2
NF-κB: nuclear factor kappa-light-chain-enhancer of activated B cells
P65: transcription factor p65
PCI: percutaneous coronary intervention
PPP: platelet poor plasma
pre-miRNA: precursor microRNA
pri-miRNA: primary microRNA
PRP: platelet rich plasma
RISC: RNA-induced silencing complex
RNAPII: RNA polymerase II
RT: room temperature
RT-qPCR: real-time quantitative polymerase chain reaction
SELE: E-selectin
TNF-α: tumor necrosis factor alpha
UPL-probe: Universal ProbeLibrary-probe
VCAM-1: vascular cell adhesion molecule-1
VSMC: vascular smooth muscle cell
