# Supplementary figures and images for: Endothelial cell activation is attenuated by everolimus via transcriptional and post-transcriptional regulatory mechanisms after drug-eluting coronary stenting
Source: PLoS One. 2018 Jun 11;13(6):e0197890. doi: 10.1371/journal.pone.0197890 (PMC5995375; doi:10.1371/journal.pone.0197890)

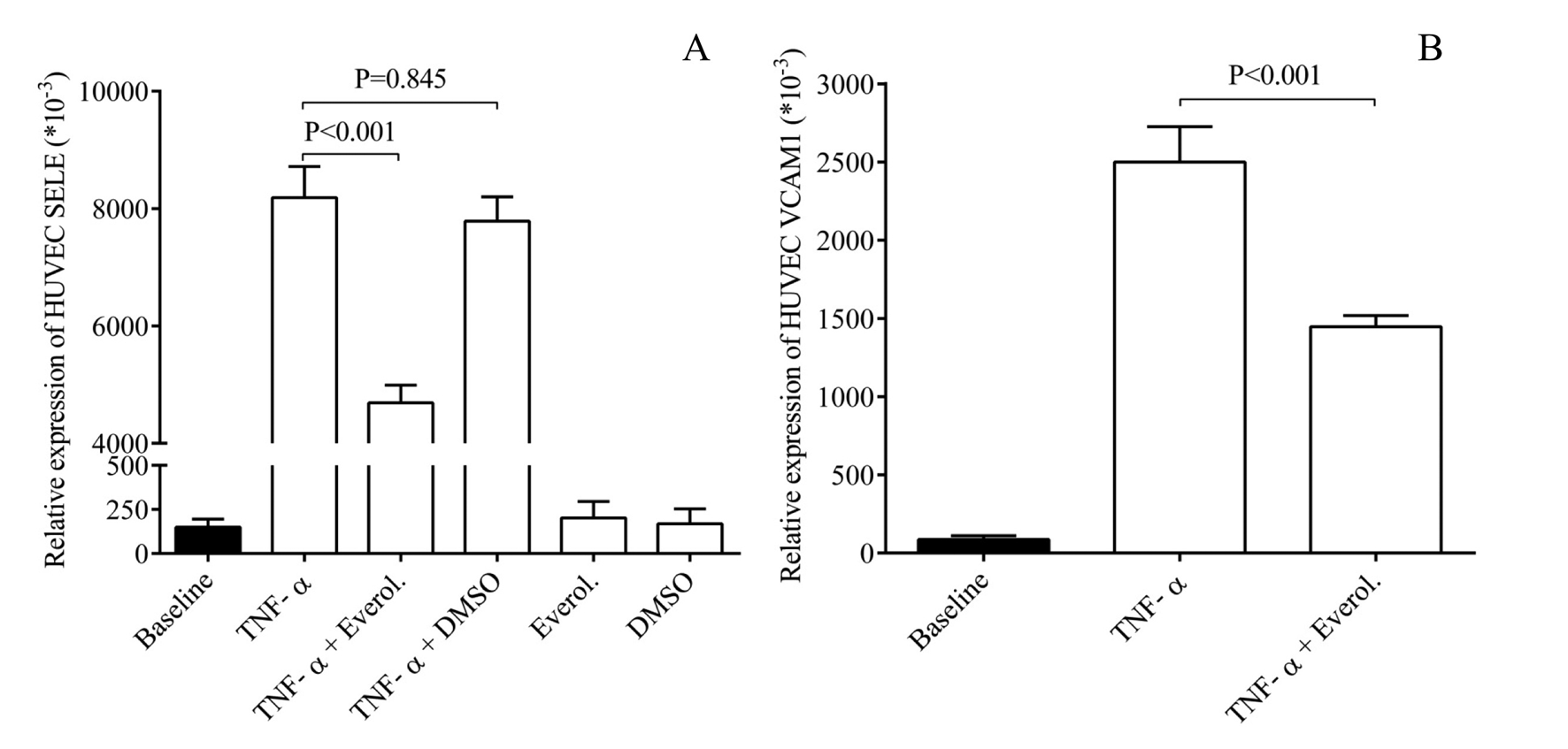

Supplement: S1 Fig — HUVECs were treated with recombinant TNF-α (100 ng/mL) for 4 hours to generate cellular inflammatory conditions. Elevated E-selectin (SELE) (A) and VCAM-1 mRNA levels (B) induced by TNF-α were downregulated by everolimus in HUVECs in vitro. Mean ± SEM, n = 4-8/group. (TIF) [file pone.0197890.s002.tif]

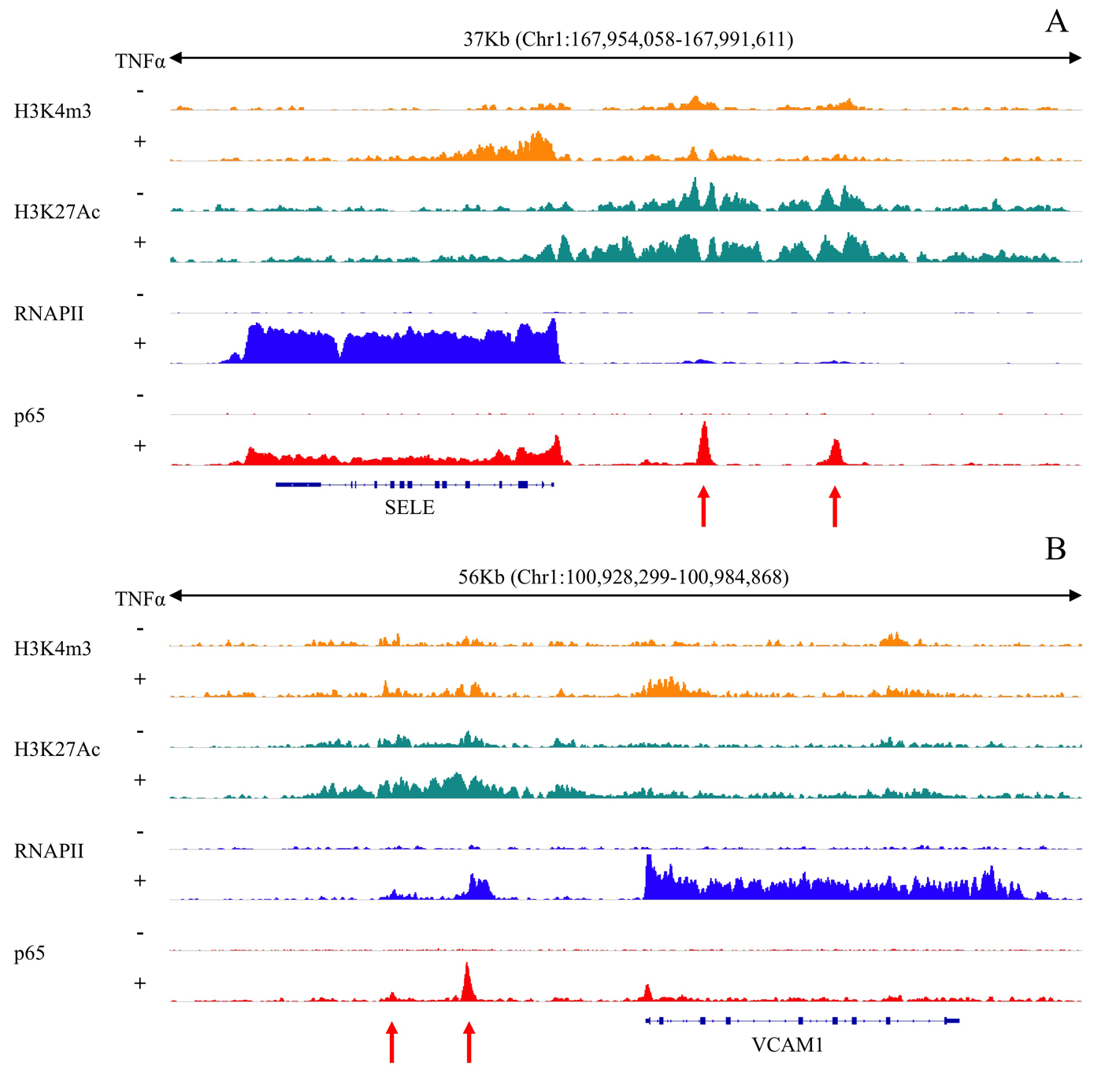

Supplement: S2 Fig — The unstimulated and TNF-α-treated HUVEC-derived publicly available NF-κB transcription factor subunit p65, RNA Polymerase II (RNAPII), active histone mark H3K27Ac, and active transcription start site mark H3K4m3-specific ChIP-seq data sets were reanalyzed. The identified TNF-α-activated p65-bound enhancers in the neighboring genomic regions of SELE (A) and VCAM1 (B) genes were indicated by red arrows. (TIF) [file pone.0197890.s003.tif]

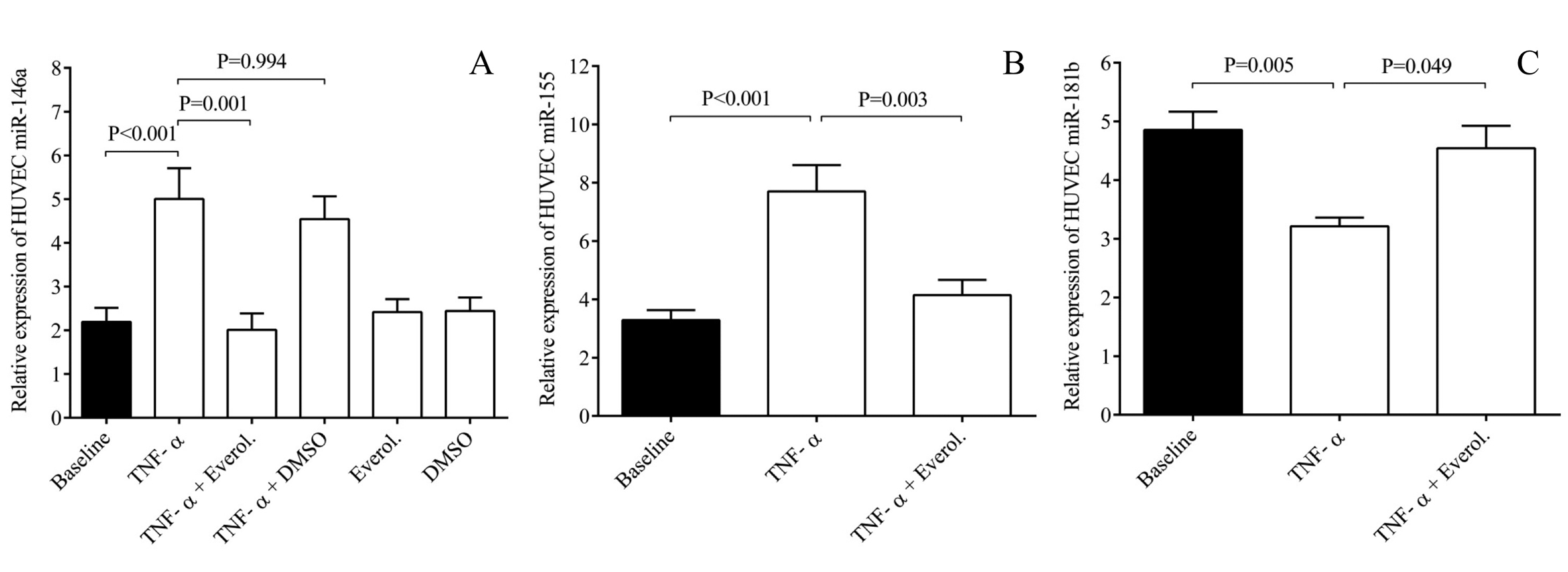

Supplement: S3 Fig — HUVECs were treated with TNF-α (100 ng/mL) with or without everolimus (0.5 μM) for 1 and 4 hours, and then these miRNA levels were quantified by RT-qPCR. Everolimus caused significantly decreased miR-146a (A) and miR-155 levels (B). The level of miR-181b (C) was downregulated by the inflammatory stimulus and the treatment with everolimus restored their expression in these EC cultures as well. Mean ± SEM, n = 4-8/group. (TIF) [file pone.0197890.s004.tif]

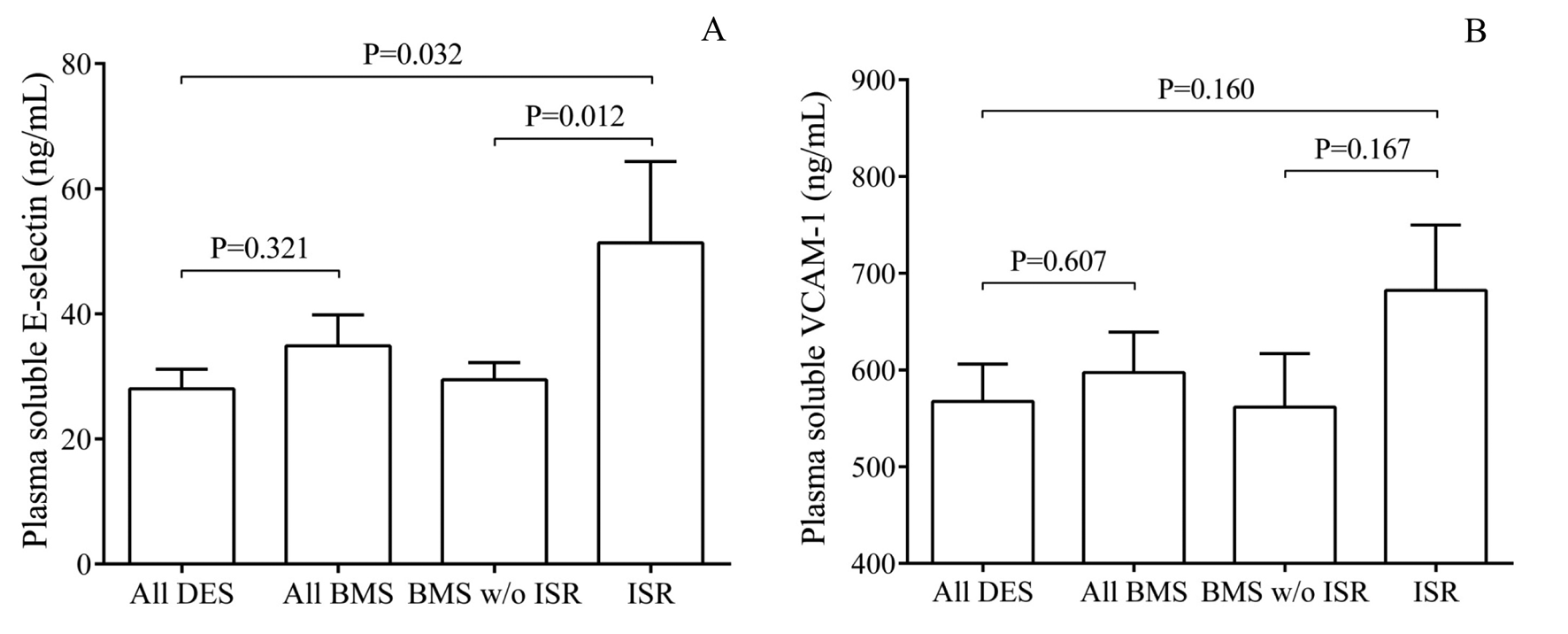

Supplement: S4 Fig — There were significantly higher E-selectin (A) and markedly elevated VCAM-1 levels in those who had ISR (n = 6) compared to other BMS (n = 22) and DES (n = 21) individuals [6]. Mean ± SEM. (TIF) [file pone.0197890.s005.tif]

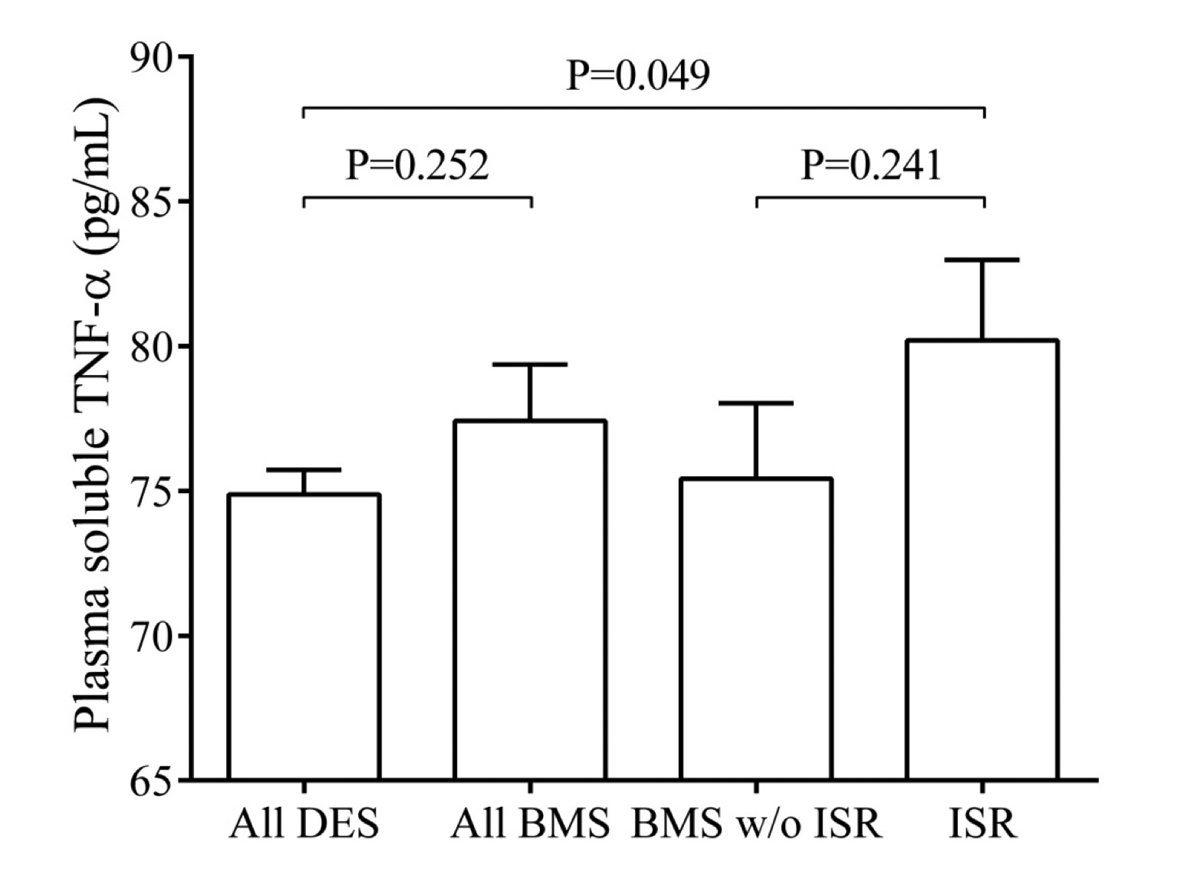

Supplement: S5 Fig — There were significantly higher plasma levels of TNF-α in those subjects who received BMS and showed ISR (n = 6) compared to individuals with DES (n = 21). Mean ± SEM. (TIF) [file pone.0197890.s006.tif]

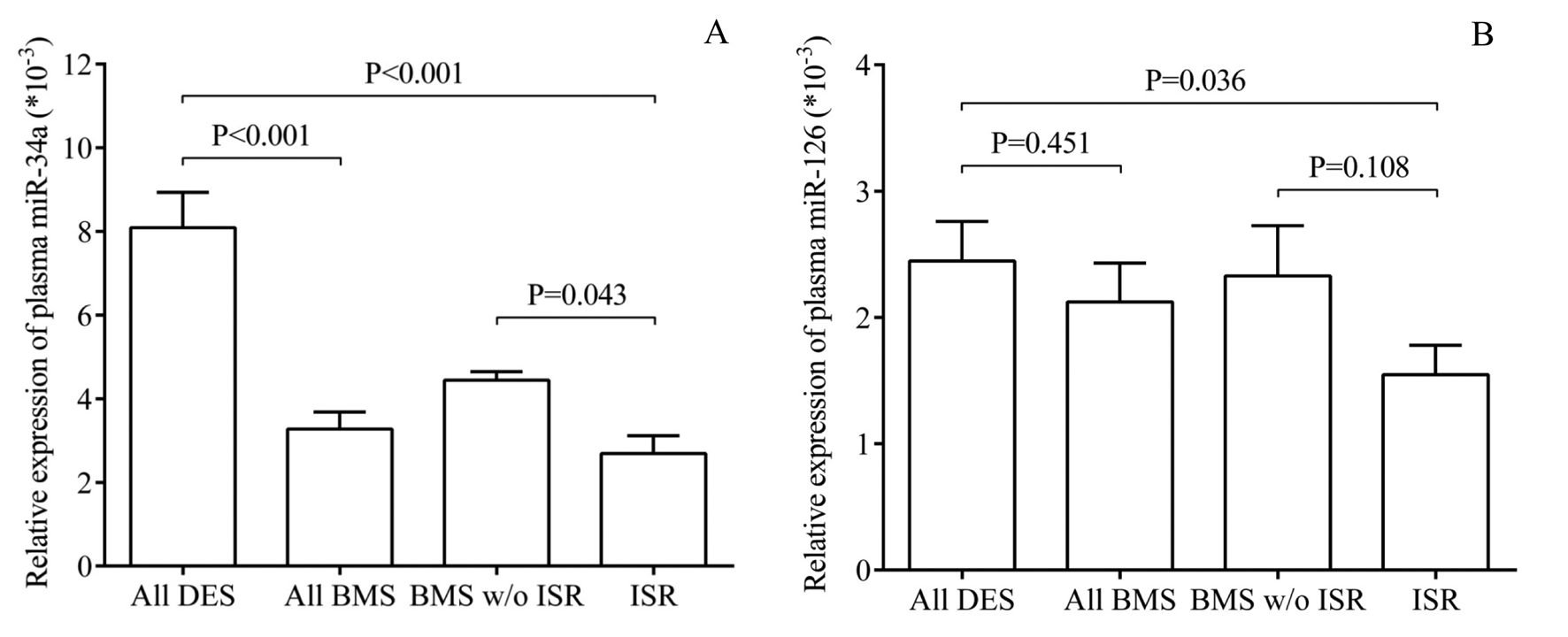

Supplement: S6 Fig — After total RNA isolation, the expression of circulating miRNAs was quantified in plasma samples by UPL-probe based stem-loop RT-qPCR assay. These miRNAs were significantly lower in those with ISR compared to BMS and DES subjects without such complication. Mean ± SEM. (All DES: n = 21, all BMS: n = 28, ISR: n = 6). (TIF) [file pone.0197890.s007.tif]
